# Acknowledgment to Reviewers of *Pediatric Reports* in 2021

**DOI:** 10.3390/pediatric14010008

**Published:** 2022-01-27

**Authors:** Pediatric Reports Editorial Office

**Affiliations:** MDPI AG, St. Alban-Anlage 66, 4052 Basel, Switzerland

Rigorous peer-reviews are the basis of high-quality academic publishing. Thanks to the great efforts of our reviewers, *Pediatric Reports* was able to maintain its standards for the high quality of its published papers. Thanks to the contribution of our reviewers, in 2021, the median time to first decision was 27 days and the median time to publication was 72 days. The editors would like to extend their gratitude and recognition to the following reviewers for their precious time and dedication, regardless of whether the papers they reviewed were finally published:
Adeyinka AdebayoJuan Bautista De SanctisAhmad MirzaJuan Carlos López-GarcíaAlessandra M. FerraroJulian AnanievAlessandra MarchesiJun-Beom ParkAlessandra Verzelloni SefKaori NakamuraAlessandro RodolicoKe-Vin ChangAlexandros PatrianakosKoji OnomotoAmutha RamadasKrishna Mohan SurapaneniAna CoelhoKristin JergerAna Isabel Álvarez-MercadoLars DietrichAna Julia García-MalinisLaura V. KlotzAnastasios KoulaouzidisLuca OppiciAndrés García-GómezMabel BladesAndrey CherstvyMalgorzata WasniewskaAnna Maria CostaMara CoyleAnna Paradowska-StolarzMāra PilmaneAnnalisa LevanteMarco CanceddaAntonio NarzisiMaria TrovatoAravind ThavamaniMarianna LeopoulouAugusto VaglioMario DiplomaticoBarbara MognettiMark W. ScerboBarbara ZaniniMarzia SegùBjörn TampeMathilde Body-MalapelCandan HizelMathilde SengoelgeCarlos Martin LlorenteMatteo Della PortaCarmen GherasimMatthias UnterhuberCarmine NovielloMattia Russel PantaloneCassandra E. HendersonMattias PaulssonChristian BeltzerMaurizio DelvecchioConcetta PolizziMelania MelisCristina MenescardiMelina ShoniDalia GobbiMelissa S.W. YamauchiDaniel J. ReidenbergMercedes Hidalgo-HerreroDavid R. RepaskeMichael W. RobertsDavide LeardiniMichele SantoroDimitri PatrikiMiguel Armengot-CarcellerDmitry VelmeshevMircea TampaDorella Del PreteMiro JukićEkhard E. ZieglerNataliya UshevaElizabeth SokolPaolo CompagnucciEnza D'AuriaPatrizia ZaramellaErnesto LevaPaul SchoenhagenEsther Moraleda SepúlvedaPedro M. RodriguesEulàlia Baselga-TorresPeter PikoEwelina Wawryk-GawdaPhil AyersFábio Saraiva FlôresPhilip TsengFátima RoquePierantonio BelliniFrancesca SciontiPierluigi MarzuilloFrancisco PerezPietro FerraraGeoffrey MadeiraPietro MaranoGeorge AnifandisPietro MuratoriGerold BesserPiotr Henryk SkarzynskiGiada GiovanniniPrashant PurohitGianluigi PastaPritmohinder S. GillGianni LazzarinProttoy HasanGiovanni Giulio ValtolinaPui Man Rosalind LaiGordana Nedic ErjavecRajan VediappanGuillermo Fernández-TardónRanjith KamityHerbert LöllgenRaquel YahyaouiHerwig CerwenkaRita PavasiniIneke Van Der HamRobert K. ClemensInês BaíaRobert PodlasekIngrida BalnyteRohan SharmaIulia-Cristina BagiuSachit AnandJacques de MouzonSanjay RathodJean KanitakisSarah Kittel-SchneiderJeanette JohnstoneSebastian BergoldJeffrey S. ForsseShinichiro MorichiJennifer M. WhittingtonShuko NojiriJimena MuciñoVerónica Martínez-LópezJo KatoVikash AgrawalJorge H. VargasWalter J. FlapperJosé Francisco López-GilXuming MaoJose María Ribera



